# Examining identity disclosure: Racial and ethnic identity amongst Multiracial/ethnic adults in the United States

**DOI:** 10.1111/hex.14083

**Published:** 2024-06-28

**Authors:** Jaimie Shaff, Janel Cubbage, Sachini Bandara, Holly C. Wilcox

**Affiliations:** ^1^ Department of Mental Health Johns Hopkins Bloomberg School of Public Health Baltimore Maryland USA

**Keywords:** ethnic and racial minorities, identity, prejudice, race factors, racial groups

## Abstract

**Objectives:**

Providing personal demographic information is routine practice in the United States, and yet, little is known about the impacts of this process. This study aims to examine the experiences and perspectives of Multiracial/ethnic adults in the United States when disclosing racial/ethnic identity.

**Methods:**

Seventeen semistructured interviews were conducted with adults identifying as Multiracial/ethnic. The Multiracial/ethnic identities of participants included Black or African American and White; Black or African American, American Indian or Alaska Native (AI/AN) and Hispanic or Latino; Black or African American and Hispanic or Latino; Black or African American and AI/AN; AI/AN and White and Asian, Native Hawaiian or Pacific Islander and White. Multiple participants reported identifying with multiple ethnic groups for any single broad category. Three identified as sexual minorities. Nine were Millennials; six were Gen X; one was Gen Z; one was Baby Boomer. Qualitative data were analyzed using staged hybrid inductive–deductive thematic analysis.

**Results:**

Disclosure of racial and ethnic identities presents a unique stressor for Multiracial/ethnic populations due to methods used to obtain data, perceived mismatch of identity and phenotype and exposure to prejudice. Social norms, constructs and movements impact the categories that a Multiracial/ethnic person indicates to external parties.

**Conclusions:**

The stress and negative feelings that Multiracial/ethnic adults face when identifying their race/ethnicity underscore the broader implications of standard demographic questions on feelings of inclusivity and visibility within a population.

**Patient or Public Contribution:**

Gathering data on individuals' racial and ethnic backgrounds is a standard practice, and yet, it can pose challenges for those who identify with multiple groups or do not see their identities reflected in the options provided. Such individuals may feel excluded or experience unfair treatment when disclosing their identity, leading to significant stress. As the frequency of this data collection increases, it is essential that the questions are posed empathetically and equitably, with a strong commitment to enhancing inclusivity throughout the process.

## 1 INTRODUCTION

Multiracial people, or people who identify with two or more racial groups, are one of the fastest‐growing demographic groups in the United States (US).^1^ Challenges with data collection, classification and management result in limited availability of data on Multiracial/ethnic populations in routine public health data reporting. Current national standards for demographic data collection, and lack of enforcement of these standards, are a limiting factor.^2^ National standards for racial and ethnic data collection set by the federal government include five categories for race (American Indian or Alaska Native [AI/AN], Asian, Black or African American, Native Hawaiian or other Pacific Islander, White, Other) and two categories for ethnicity (Hispanic or Latino, not Hispanic or Latino).^2^ Despite the existence of these national standards, multiple factors, including limitations of data infrastructure, result in incomplete data.^3^

Racial categories are socially constructed and have changed throughout history.^4^, ^5^ When the US Census Bureau added the ability to select more than one race in the 2000 Census, there was much debate and controversy about the need, legality, or utility of quantifying the Multiracial population.^6^ The lack of implementation of the national standard to ensure that individuals can select multiple categories continues to inhibit data collection efforts. The US Federal Government has recently revitalized efforts to update federal standards for racial and ethnic data collection, potentially transforming the methodology and available categories nationwide.^7^ There has been some consensus within scientific communities that there should be a minimum of seven distinct racial groups due to unique racialized experiences in the United States, and this is reflected in the drafts for the updated 2030 Census format, which no longer asks separately about Hispanic ethnicity but includes all of seven groups in one question.^4^ Improvements that allow for greater disaggregation within these seven categories are encouraged.

The health impacts connected to the prejudice and stigma experienced by minoritized populations are often referred to as *minority stress*, a conceptual framework that underpins a large volume of health research conducted amongst sexual minorities.^8^, ^9^, ^10^, ^11^, ^12^ Although much of the literature using minority‐stress‐related conceptual frameworks is focused on sexual minorities, the minority stress framework is theoretically applicable to any stigmatized minority group; a growing body of evidence demonstrates the connection between race‐related stress and health.^13^

The modified integrative mediation framework developed by Timmins et al.^14^ builds from Meyer's^10^ minority stress model and Hatzenbuehler's^15^ subsequent expansion in 2009 and summarizes the impact of demographic variables, environment, minority stressors, minority group stressors, and psychological processes on mental health.^10^, ^14^, ^15^ This framework includes a domain for the disclosure of minoritized identity, specifically the concept of ‘outness’ for minoritized sexual identities, finding complex relationships between identity disclosure, other known stressors within minoritized communities, and mental health outcomes.^14^ The US's complex history with Multiracialism, including the debate and controversy surrounding the changes made for Census 2000 and recent surges in antimiscegenation movements, raises the question of the impact of disclosure of racial/ethnic identity for this minoritized population.^7^, ^16^ Recent data identify Multiracial populations in the United States as having some of the highest prevalence of mental health concerns of any other racial/ethnic group, and it is critical that the scientific field better understand potential risk and protective factors for this rapidly growing population.^16^, ^17^, ^18^

Racial and ethnic identity is a demographic variable that is routinely requested. Given increasing attention on growing health inequities, known data‐collection challenges that surfaced during the COVID‐19 pandemic, and renewed attention on these variables by the federal government, it is expected that racial/ethnic data collection will continue to grow.^3^, ^7^, ^19^ Recent studies have explored impacts of Census 2020 racial options within populations of Multiracial young adults and describe the different ways in which Multiracial young adults self‐disclose their racial/ethnic identity for the Census along with associations with self‐esteem.^20^, ^21^ However, little is known about views, processes and conceptualization of self‐reporting race and ethnicity, the impact of forced‐choice categories, or experiences of stigma related to disclosure of minority status for older Multiracial/ethnic adult populations, particularly Multiracial/ethnic populations experiencing mental health concerns.^22^

Given the theoretical connection between disclosure of a minoritized identity and mental health‐related outcomes, and urgent need to address mental health within this population, this study aims to investigate aspects of racial/ethnic identity disclosure within a population of Multiracial adults in the United States that endorsed clinically significant symptoms of depression, anxiety, PTSD, or suicidal thoughts and behaviours. This study focuses on the following key research questions: (1) What experiences have Multiracial/ethnic people had when self‐disclosing their racial/ethnic identity throughout their lifetime? (2) What sentiments do Multiracial/ethnic people have regarding options provided for race/ethnicity? (3) What changes, if any, have Multiracial/ethnic people made when providing racial/ethnic data and why?

## 2 METHODS

Semistructured interviews were conducted via videoconference or audio call from December 2022 to January 2023 with 17 US adults 18 or older who identified as Multiracial and/or multiethnic. To the authors' knowledge, most public health entities and scientific research studies do not incorporate Hispanic and Latino populations within their definition of Multiracial. As this study includes Hispanic and Latino populations, populations identifying as ‘Middle Eastern or North African,’ and populations with multiple ethnic backgrounds in any single racial category, this study refers to the sample population broadly as Multiracial and/or multiethnic. This study is developed based on the principles of participatory action research, was developed and led by a member of the community being studied and includes the target community, Multiracial/ethnic people, in all stages of the research including study design, instrument development, data collection and analysis and interpretation of findings.^23^, ^24^, ^25^, ^26^

### 2.1 Participant recruitment and sampling approach

Study participants were recruited from a pool of respondents from a cross‐sectional survey (*N* = 347) conducted in February–June 2022 that endorsed at least one symptom of a mental health condition and agreed to participate in a future qualitative study.^16^ Endorsement of mental health conditions was established using cutoffs for clinically significant symptoms of anxiety (Generalized Anxiety Disorder‐2 score ≥ 3), depression (Patient Health Questionnaire‐9 score ≥ 10), posttraumatic stress disorder (PTSD; PTSD Checklist for DSM‐5 score ≥ 33), or endorsement of any of the five measures for suicidal thoughts or behaviours. Recruitment was conducted by first randomly selecting potential participants from the established pool and then conducting targeted outreach to random selections of participants who reported minoritized identities underrepresented by the initial round of selection. The final sample size was established when the research team determined that thematic saturation had been reached, or when new codes/themes no longer emerged, and efforts to improve inclusion of intersectional identities had been exhausted.^27^, ^28^, ^29^

The Johns Hopkins University Institutional Review Board (IRB) approved this study. Interviews were conducted by a single member of the research team and lasted a maximum of 60 min. To participate in the qualitative interview, participants provided oral consent after being read the IRB‐approved oral consent script by the interviewer. Participants verbally consented to participating in the interview and consented to having their interview recorded. Audio recording was not a requirement to participate in the interview. For participating in the qualitative interview, participants received a $25 Amazon gift card as a small token of appreciation.

### 2.2 Instrument development

An interview guide was developed in collaboration with members of the Multiracial/ethnic community. The interview explored areas of ethnic identity and identity alignment underassessed in quantitative research. Three individuals who identified as Multiracial and/or multiethnic and two individuals who identified with other communities of colour piloted the interview guide to refine the language of the questions and ensure inclusion of thematic areas of interest. The interview explored experiences of self‐identifying race and ethnicity for standard items on surveys, standardized tests, and medical documentation to better understand the personal impact of standard and nonstandard classification structures. Participants were asked to describe experiences self‐identifying their race and/or ethnicity and any changes that they have made during their lifetime in how they self‐identify their racial and ethnic background.

### 2.3 Data analysis

Interview transcripts were analyzed using staged hybrid inductive–deductive thematic analysis using Dedoose, a collaborative software application developed for managing, analyzing, and presenting qualitative and mixed‐methods research data.^30^ The research team created an initial codebook based on interview questions, which were based on the study questions of interest, a priori knowledge on the subject, and notes from the interviews. Three members of the study team piloted the codebook by coding two random transcripts from the first five interviews. Afterwards, the team met to discuss interpretation of the codes, adjust codes and definitions, and note any additional codes with the goal of capturing themes and subthemes that would inform the main research questions. Two members of the study team then double‐coded two transcripts and met to discuss any adjustments to the codebook. After these steps, the final codebook was used by a primary coder who identified as Multiracial/ethnic to code the remaining transcripts, which was then reviewed by a secondary coder who identified as Multiracial/ethnic.

After coding was completed, the study team analyzed narratives using thematic analysis and identified domains, primary themes, and subthemes related to the research questions. Common sentiments, domains, and themes were summarized and accompanied by illustrative quotes (Table 1). Key findings were reviewed by the analytic team. Subgroup analyses were conducted amongst subsets by racial and ethnic composition of the participants, gender identity, sexual orientation, and age. As part of the participatory approach, preliminary results were shared back with the interview participants, who were provided an opportunity to review the results and provide additional feedback and comments. This approach provides a continuous feedback loop and an opportunity for participants to remain included in the research. This opportunity to provide additional feedback was completely optional, and feedback received was anonymous and not tied to the individual participant. Feedback received was incorporated into the findings.

**Table 1:** Qualitative codebook.

Domain	Parent code	Child code	Grandchild code	Description	Sample quotation
Factors that impact disclosure of race/ethnicity and strength in ethnic and racial identity				Experiences, thoughts and feelings related to the way they self‐disclose their racial and ethnic identity to external parties.	‘it's always a little stressful and then having to pick one or the other is something that I dread. And then it's also that that weird thing of well, because I'm somewhat Black, then I'm definitely Black’.
	Sentiments of options for race/ethnicity that are available on forms			The person shares experiences and feelings related to the options for race/ethnicity made available to them on forms and documents. May include feelings related to the lack of standardization of options, or having to think about making a choice.	‘I just think that this world is built still on monoracial social constructions, right. Like when I see these, like, clear cut boxes that we're meant to fit in and, like, well what do you do. Sometimes it's pretty defeatist’.
	Impact of alignment of identity with social norms in selection process			Person considers how their identity fits with or is perceived by society when deciding how to indicate their racial/ethnic background.	‘life has been a journey of figuring out my identity. And as I got through college, it was the place of OK, I am, you know, I am staunchly Multiracial and then over the last few years, seeing social justice movements, the stuff during 2020, George Floyd Times and stuff and it's kind of choosing that point of not siding with the oppressor’.
		Social norms, constructs and movements impact the racial and ethnic categories that a Multiracial and/or multiethnic person indicates to external parties		The person describes the impact of social norms, constructs, movement and culture on the way they self‐disclose their racial and ethnic identity.	‘Often it is, choose one. And then historically you sometimes don't wanna choose either because you're afraid of what would happen or what type of service you could get’.
		Sentiments of belonging and rejection		The person describes experiences and feelings where they felt they could or could not present and/or be visible as their full racial/ethnic self.	‘the problem with that is it's a battle for me because we are native, but we're not recognized as a tribe and there's politics behind that …. filling out a form—it's like which part of me kind of dies, you know, just for this simple classification’.
			Impact of perceived mismatch between physical presentation and characteristics such as name and/or cultural upbringing	A person shares impacts from the way others perceive the fit between their true racial and ethnic identity and visible factors such as appearance and name.	‘I wouldn't mind it so much if the person who usually greets me isn't so visibly disappointed…. They're just never accepting of that, whatever you are, you're the person that they're there to see’.
	Feeling of being or being perceived as dishonest or truthful			A person describes experiences of selecting categories that match and do not match their full racial and ethnic identity and feeling or being perceived as dishonest or truthful.	‘The biggest stressor in those moments was not … or feeling kind of dishonest. Or feeling like I wasn't true to myself’.

### 2.4 Results

A total of 17 interviews were conducted and a codebook was developed to elucidate domains, themes, and subthemes (Table 1). Eleven participants identified as female; four as male; and two as gender‐expansive. Fourteen of the participants identified as straight and three as bisexual or other sexuality. Nine participants were Millennials; six were Gen X; one was Gen Z; and one was Baby Boomer. Twelve of the Multiracial/ethnic interviewees selected the racial/ethnic category for White; eight for Asian; seven for Black or African American; four for AI/AN; and two for Hispanic or Latino. The Multiracial/ethnic identities of participants included Black or African American and White; Black or African American, AI/AN, and Hispanic or Latino; Black or African American and Hispanic or Latino; Black or African American and AI/AN; AI/AN and White; and Asian, Native Hawaiian or Pacific Islander and White. Multiple participants reported identifying with multiple ethnic groups for any single broad category.

### 2.5 Sentiments of options for race/ethnicity that are available on forms

Standard demographic questions about race and ethnicity often posed challenges for Multiracial/ethnic individuals. Participants recalled the stress induced by such questions in childhood, especially during standardized testing. The requirement to select a single racial category evoked negative emotions, including shame and a sense of self‐denial. The inability to acknowledge their full identity made them feel dishonest. Conversely, the option to select multiple categories was met with positive reactions such as validation and pride. However, forms that still limit choice to one category can elicit feelings of identity death or erasure.

It was great to kind of be validated of I am a thing that that can exist and not just a ‘other’.

It's like, which part of me kind of dies just for this simple classification.

Inconsistencies in racial and ethnic data collection have affected Multiracial/ethnic individuals. Changes, such as the merging or removal of categories, have led to feelings of erasure. Entities that collect data and allow only one racial or ethnic choice are viewed as outdated or incorrect, influencing the willingness of Multiracial/ethnic people to provide information. Some reported opting to bypass these questions altogether, feeling that the data collector does not deserve their data. Participants found broader categories to be less meaningful and unable to reflect their true identities or cultural experiences. The standard two‐question format, which separates ‘Hispanic’ as an ethnicity, was seen as confusing, with lack of clarity about its definition and the reason for exclusion of other ethnicities. Additionally, those whose racial or ethnic backgrounds did not match their country of origin (i.e., identify as having origins in China and the United Kingdom but born and raised in Malaysia) reported facing extra difficulties in self‐identification. Participants also highlighted the need to disaggregate within the larger categories, given the vastly different experiences of these groups in the United States Those who had taken DNA ancestry tests also struggled with the results' implications for their self‐identification.

I just think that this world is built still on monoracial social constructions, right. Like when I see these, like, clear cut boxes that we're meant to fit in and, like, well what do you do. Sometimes it's pretty defeatist.

Multiracial/ethnic participants sought clearer explanations on the usage and purpose of race and ethnicity data, which would inform them about the significance of their responses. Participants noted that one's racial and ethnic identity might not align with their cultural upbringing, and the reason for data collection can impact their decisions to self‐disclose their identities. Participants advocated for more inclusive data collection methods, especially as society evolves and data collection technologies improve. This was especially important for individuals who disclosed being neurodiverse, who reported that they often struggle to fit into existing categories or understand the use of the data.

My biggest hope is that it does let you check multiple ones and being able to combine things because that really is my identity is that it's multiple places. So. Yeah, it's always a little stressful and then having to pick one or the other is something that I dread.

### 2.6 Social norms, constructs and movements impact the racial and ethnic categories that a Multiracial and/or multiethnic person indicates to external parties

Participants reflected on how societal norms and movements influence their racial or ethnic identification on forms or documents. Participants expressed fatigue over repeatedly having to justify their racial or ethnic identity, the origins of their name and their cultural background. As children, they were conscious of how data collectors might perceive their choices in relation to their names or appearances. This awareness often led them to select the race or ethnicity that matched their name or appearance due to concerns about being seen as dishonest. Participants also noted the surprise of others when their in‐person appearance did not match expectations based on their name or resume. For instance, Multiracial/ethnic individuals whose names were typically assigned to White people reported choosing the category ‘White’ to avoid scepticism from data collectors and have contemplated changing their name to better align with their true racial/ethnic identity and reduce potential bias.

To White people I'm not White, but to Asians I'm not Asian.

The evolving social recognition and acceptance of Multiracial/ethnic individuals influences their willingness to disclose their identities. Participants from various backgrounds referenced the historical ‘one‐drop rule’ in the United States, which deemed individuals with any Black ancestry as Black, and noted its unclear application to other minorities. The racial and ethnic makeup of their community also impacted participants' comfort in disclosing their identities; in homogeneous areas, they often hesitated due to safety concerns. Participants reported weighing the risk of discrimination when selecting racial or ethnic categories, often choosing the one that minimized potential prejudice. For instance, some might omit answering the race question or select ‘other’ when faced with anticipated bias, such as in health care settings, preferring to confront discrimination in person rather than on paper.

Often it is, choose one. And then historically you sometimes don't wanna choose either because you're afraid of what would happen or what type of service you could get.

Participants have found it easier to embrace their Multiracial/ethnic identities amid growing acceptance from monoracial groups and the decline in the use of derogatory terms such as ‘mulatto,’ ‘mutt,’ ‘half breed’ and ‘love child’. They noted a positive change in popular culture's portrayal of mixed‐race individuals, moving from negative depictions to more empowering representations by Multiracial public figures. During significant social movements addressing racial violence, such as the George Floyd protests in 2020 and the Vincent Chin case in 1982, those sharing racial or ethnic identities with the victims felt a stronger urge to acknowledge these identities on forms, aiming to bolster visibility for affected communities. These societal changes have paved the way for Multiracial/ethnic individuals to fully acknowledge their identities, though they still encounter instances of rejection from monoracial groups.

… it gets tricky because if you claim a mixed identity, a lot of folks, especially within the Black community, will read that as—you're rejecting your Blackness—or—do you think you're better than anyone else because of your light skin, all that colorism is real. But it's also my reality.

## 3 DISCUSSION

This study highlights challenges experienced by Multiracial/ethnic people when providing racial and ethnic demographic information on standard documents and forms. Extreme feelings of stress, despair, and identity death are connected to providing this information and can be exacerbated by the way the question is asked and the categories made available. The lack of standardization for this demographic question and how often the data are collected may create a compounding, unrelenting stressor unique to this Multiracial/ethnic people, with clear implications on health.^31^, ^32^ Although federal standards now permit the selection of multiple races, the enforcement of this option is inadequate, and the current categories fail to accurately reflect the diversity within racial and ethnic groups. For instance, ‘AI/AN’ represents over 500 tribes across two continents, ‘Asian’ covers more than 48 countries, and ‘Black or African American’ refers to both skin colour and African lineage, thereby oversimplifying vast diversities.^33^, ^34^, ^35^, ^36^ The Hispanic and Latino communities also face challenges, as the current separation of race and ethnicity in standards conceals their racial diversity, and there is a lack of consistency in analytic approaches often resulting in the exclusion of this entire population.^7^, ^37^ Additionally, people of Middle Eastern and North African (MENA) descent are often inaccurately prompted to select ‘Other’ or ‘White’ categories that fail to represent this group's complex identity.^38^ Studies have noted the deleterious impact that erasure of the MENA community has had on the mental and physical well‐being of these communities, as well as the public health importance of including this broad racial category in data collection.^39^

The extreme negative sentiments and stress that Multiracial/ethnic adults experienced from childhood through adulthood related to answering the demographic question are troubling, as it suggests that this experience could be potentially traumatic and may have implications for child development. To the authors' knowledge, this has not yet been explored in traumatic stress or child development research. Although associations between minority stress and mental health have been well established for monoracial populations, evidence to describe the stressors and associated impacts experienced by Multiracial/ethnic populations is lacking.^10^, ^11^, ^13^ Timmins' modified integrative mediation framework, developed to test the impact of potential stressors amongst lesbian, gay, and bisexual individuals, hypothesizes disclosure of minority status to be negatively associated with self‐stigma, active concealment, and expectations of rejection, but positively associated with prejudice events.^14^ This study provides evidence to suggest a similar relationship for disclosure of Multiracial/ethnic identities that needs to be further explored in scientific research. As studies have demonstrated the differences in the decision‐making process amongst neurodiverse populations, the findings from this study suggest that additional stress may be experienced by neurodiverse Multiracial/ethnic populations when deciding how to self‐report race and ethnicity, which warrants further research.^40^, ^41^

This study also describes unique aspects of prejudice events specifically related to providing racial and ethnic demographic information that may not be currently captured by commonly used frameworks.^42^, ^43^, ^44^ For example, the perception that data owners will ascribe dishonesty to a person if racial and ethnic information does not align with social norms could be supported by reactions that Multiracial/ethnic people described when someone met them in person for the first time. This investigation was beyond the scope of this study, and further research into how monoracial individuals perceive Multiracial/ethnic people, as well as opportunities to reduce bias from monoracial communities, is recommended.

The findings from this study provide evidence to support the value of representation in popular culture and in reducing social acceptance of harmful stereotypes and language. This study suggests that this supports Multiracial/ethnic people to embrace their ethnic identity and integrate their multicultural identities, while also increasing acceptance of Multiracial/ethnic people from predominantly monoracial groups. As the literature suggests that belonging to social groups, strength in ethnic identity, and multicultural ethnic identity integration can serve to protect against mental health issues, we recommend continued expansion in these areas.^45^, ^46^, ^47^, ^48^

### 3.1 Strengths and limitations

This study has several strengths. As the study was conducted by and for Multiracial/ethnic populations, the study included questions that explore identity‐related constructs unique to Multiracial/ethnic populations. During piloting of the study, questions were replaced or modified when at least two of three members of piloting team understood the question in a different way. The sample obtained for the study was robust, and there were few modifications to the codebook after the seventh interview. Additionally, the sample included diversity within the Multiracial/ethnic community, allowing the study team to explore aspects across racial and ethnic identity, age, gender, and sexuality.

There are several limitations to this study. As inclusion criteria for this study required participants to have completed a previously administered online cross‐sectional study, endorse at least one mental health symptom and agree to participate in a follow‐up interview, the pool of potential respondents was limited and results will have limited generalizability. Subgroup analyses were dependent on the demographics of the participants of this phase of the study. As the study was developed, conducted and analyzed by Multiracial/ethnic adults, this may have influenced the questions that were asked, the way participants responded to the questions and how the data were analyzed. As this study uses current constructs for racial/ethnic identities in the United States, it is important to recognize that this approach to gathering racial/ethnic background does not fully or adequately capture the rich diversity of the study population, nor the language used by each individual participant to describe their background.

## 4 IMPLICATIONS FOR POLICY AND PRACTICE

It is critical for the public health community to develop methodological processes and engage in thorough research to ensure that the diverse health needs of Multiracial/ethnic populations, and other minoritized populations underrepresented in research, are visible and addressed. This study supports findings by Atkins and Minniear on the experiences of Multiracial young adults with racial/ethnic identity options established by the federal government for the Census, adding to the literature by expanding these findings to older Multiracial populations inclusive of Hispanic/Latino people.^20^, ^21^ Additionally, this study adds evidence to support findings by Timmins et al. on the health impacts of minoritized identity disclosure and expands those findings to another minoritized identity.^14^ These findings highlight the necessity for further research and investigation into the stressors and prejudice events experienced by Multiracial/ethnic people related to racial/ethnic identity disclosure and associated impacts on health. They also provide evidence to suggest that the domain established by Timmins et al. for ‘outness’ could be considered more broadly to other minoritized identities socially required or expected to disclose their minoritized status.

It is crucial that we evaluate and improve systems that monitor population health and structural factors affecting the public health workforce's ability to recognize and respond to the health needs of all our populations. One such structural factor is with the national standards for racial and ethnic data collection established by the federal government.^2^ As the Federal Government sets these standards and is responsible for ensuring their implementation, this study supports efforts to expand inclusivity and ensure proper application of these standards. This is an important step to ensure that electronic record systems can adequately capture and report against these standards and will greatly support the public health community in improving data quality and health outcomes.^3^, ^49^ However, it is important to note that, as a social construct, race is sensitive to modulating strength in ethnic identity and evolving social norms for selecting racial or ethnic categories, which may result in limited comparability of data over time and require a more flexible approach to data classification.^22^, ^46^, ^50^ With this in mind, we recommend that there be consideration of the opportunities to leverage advances in technology to create more flexible standards and reduce the burden on Multiracial/ethnic people to categorize themselves into available options. Further, there is a need for a tailored approach that is responsive to the various data‐related concerns and experiences of different communities. For example, it is critical that approaches be responsive to tribal nations’ capacity to be involved in and lead these processes, given that the political status of tribal nations and tribal citizens is separate from race/ethnicity.^35^

This study also highlights a need to build the capacity of educators, health professionals, and employers in supporting Multiracial/ethnic children and adults as they navigate these demographic questions. The stress experienced by respondents should be weighed against data management needs, with respondent health and safety as the priority. For example, testing programs might consider asking demographic questions postexamination to mitigate their impact and train proctors to better assist students with these queries. Entities capable of providing a long list of countries for participants to select their identity from, or providing an open‐text answer field, could consider building this into their data systems. Moreover, entities that collect racial and ethnic data should provide clear explanations regarding the purpose and use of the data collected.

Data‐driven resource allocation is limited by the availability of data. While this study focused on racial/ethnic identity, the findings and the outcomes have broader implications for data collection on marginalized and minoritized identities. It is essential to recognize and address the potential adverse effects on these individuals and communities, striving to minimize harm. In areas where marginalized identities are especially vulnerable to hate, bias, and prejudice, it is critical for public health authorities and other data collectors to place safety at the forefront. If collecting demographic data is unsafe or risks harm to individuals, then alternative strategies must be used to ensure that those communities receive sufficient resources. This study calls for a community‐responsive, inclusive, and safety‐focused approach to data collection that respects the risks faced by marginalized groups. Protecting these communities and ensuring equitable resource distribution are not just data challenges but also a moral imperative that requires immediate and careful attention.

## AUTHOR CONTRIBUTIONS

**Jaimie Shaff**: Conceptualization; investigation; funding acquisition; writing—original draft; methodology; formal analysis; data curation; project administration; supervision; software; resources; visualization; validation. **Janel Cubbage**: Writing—review and editing; formal analysis; validation. **Sachini Bandara**: Data curation; writing—review and editing; methodology; validation. **Holly C. Wilcox**: Investigation; funding acquisition; writing—review and editing; methodology; project administration; supervision; resources.

## CONFLICT OF INTEREST STATEMENT

The authors declare no conflict of interest.

## ETHICS STATEMENT

This study was approved by the Johns Hopkins University Institutional Review Board, IRB# 18482. Informed consent was obtained verbally before participation.

## Data Availability

The participants of this study did not give written consent for their data to be shared publicly. Due to the sensitive nature of the research, supporting data are not available.

## References

[1] United States Census Bureau . 2020 Census illuminates racial and ethnic composition of the country. 2021. Accessed February 12, 2024. https://www.census.gov/library/stories/2021/08/improved-race-ethnicity-measures-reveal-united-states-population-much-more-multiracial.html

[2] Office of Management and Budget . Revisions to the standards for the classification of federal data on race and ethnicity. 1997. Accessed February 12, 2024. https://www.govinfo.gov/content/pkg/FR-1997-10-30/pdf/97-28653.pdf

[3] Council of State and Territorial Epidemiologists . Addressing gaps in public health reporting of race and ethnicity data for COVID‐19: findings & recommendations among 45 state & local health departments. 2024. Accessed February 12, 2024. https://preparedness.cste.org/wp-content/uploads/2022/04/RaceEthnicityData_FINAL.pdf

[4] Atkin AL , Christophe NK , Stein GL , Gabriel AK , Lee RM . Race terminology in the field of psychology: acknowledging the growing multiracial population in the US. Am Psychol. 2022;77(3):381‐393. 10.1037/amp0000975 35254853 PMC9316411

[5] Braveman P , Parker Dominguez T . Abandon “Race.” Focus on racism. Front Public Health. 2021;9:689462. 10.3389/fpubh.2021.689462 34557466 PMC8452910

[6] Perlmann J , Waters MC . New Race Question, How the Census Counts Multiracial Individuals. Russell Sage Foundation; 2002. https://www.jstor.org/stable/10.7758/9781610444477

[7] Artiga S , Pillai D . Proposed changes to federal standards for collecting and reporting race/ethnicity data: what are they and why do they matter? KFF. 2023. Accessed February 12, 2024. https://www.kff.org/policy-watch/proposed-changes-to-federal-standards-for-collecting-and-reporting-race-ethnicity-data-what-are-they-and-why-do-they-matter/

[8] de Lange J , Baams L , van Bergen DD , Bos HMW , Bosker RJ . Minority stress and suicidal ideation and suicide attempts among LGBT adolescents and young adults: a meta‐analysis. LGBT Health. 2022;9(4):222‐237. 10.1089/lgbt.2021.0106 35319281

[9] Flentje A , Heck NC , Brennan JM , Meyer IH . The relationship between minority stress and biological outcomes: a systematic review. J Behav Med. 2020;43(5):673‐694. 10.1007/s10865-019-00120-6 31863268 PMC7430236

[10] Meyer IH . Prejudice, social stress, and mental health in lesbian, gay, and bisexual populations: conceptual issues and research evidence. Psychol Bull. 2003;129(5):674‐697. 10.1037/0033-2909.129.5.674 12956539 PMC2072932

[11] Pellicane MJ , Ciesla JA . Associations between minority stress, depression, and suicidal ideation and attempts in transgender and gender diverse (TGD) individuals: systematic review and meta‐analysis. Clin Psychol Rev. 2022;91:102113. 10.1016/j.cpr.2021.102113 34973649

[12] Wolford‐Clevenger C , Hill SV , Cropsey K . Correlates of tobacco and nicotine use among transgender and gender diverse people: a systematic review guided by the minority stress model. Nicotine Tobacco Res. 2022;24(4):444‐452. 10.1093/ntr/ntab159 PMC11494233 34375426

[13] Williams DR . Stress and the mental health of populations of color: advancing our understanding of race‐related stressors. J Health Soc Behav. 2018;59(4):466‐485. 10.1177/0022146518814251 30484715 PMC6532404

[14] Timmins L , Rimes KA , Rahman Q . Minority stressors, rumination, and psychological distress in lesbian, gay, and bisexual individuals. Arch Sex Behav. 2020;49(2):661‐680. 10.1007/s10508-019-01502-2 31332645 PMC7031186

[15] Hatzenbuehler ML . How does sexual minority stigma “get under the skin”? A psychological mediation framework. Psychol Bull. 2009;135(5):707‐730. 10.1037/a0016441 19702379 PMC2789474

[16] Shaff J , Wang X , Cubbage J , Bandara S , Wilcox HC . Mental health and Multiracial/ethnic adults in the United States: a mixed methods participatory action investigation. Front Public Health. 2024;11:1286137. 10.3389/fpubh.2023.1286137 38274534 PMC10808380

[17] SAMHSA . 2022 National Survey on Drug Use and Health (NSDUH) releases. 2024. Accessed February 12, 2024. https://www.samhsa.gov/data/release/2022-national-survey-drug-use-and-health-nsduh-releases#detailed-tables

[18] The Trevor Project . The mental health and well‐being of Multiracial LGBTQ youth. August 11, 2022. Accessed February 12, 2024. https://www.thetrevorproject.org/research-briefs/the-mental-health-and-well-being-of-multiracial-lgbtq-youth-aug-2022/

[19] Bailey ZD , Krieger N , Agénor M , Graves J , Linos N , Bassett MT . Structural racism and health inequities in the USA: evidence and interventions. Lancet. 2017;389(10077):1453‐1463. 10.1016/S0140-6736(17)30569-X 28402827

[20] Atkin AL , Minniear M . An exploratory mixed methods study of multiracial Americans' race choices on the 2020 census. Cultur Divers Ethnic Minor Psychol. 2023;29(3):406‐417. 10.1037/cdp0000582 36931844

[21] Minniear M , Atkin AL . Exploring Multiracial identity, demographics, and the first period identity crisis: the role of the 2020 United States Census in promoting monocentric norms. J Appl Commun Res. 2023;51(1):37‐54. 10.1080/00909882.2022.2107401

[22] Charmaraman L , Woo M , Quach A , Erkut S . How have researchers studied multiracial populations: a content and methodological review of 20 years of research. Cultur Divers Ethnic Minor Psychol. 2014;20(3):336‐352. 10.1037/a0035437 25045946 PMC4106007

[23] Baum FE . Power and glory: applying participatory action research in public health. Gac Sanit. 2016;30(6):405‐407. 10.1016/j.gaceta.2016.05.014 27491431

[24] Israel BA , Schulz AJ , Parker EA , Becker AB . Review of community‐based research: assessing partnership approaches to improve public health. Annu Rev Public Health. 1998;19:173‐202. 10.1146/annurev.publhealth.19.1.173 9611617

[25] Minkler M . Using participatory action research to build healthy communities. Public Health Rep. 2000;115:191‐197. 10.1093/phr/115.2.191 10968753 PMC1308710

[26] Thompson B , Molina Y , Viswanath K , Warnecke R , Prelip ML . Strategies to empower communities to reduce health disparities. Health Aff. 2016;35(8):1424‐1428. 10.1377/hlthaff.2015.1364 PMC5554943 27503967

[27] ASPE . Advancing equity by incorporating intersectionality in research and analysis. 2022. Accessed February 12, 2024. https://aspe.hhs.gov/sites/default/files/documents/80123172bbe4458a06259535dc3fcfc3/Intresectionality-Resrch-Anlysis.pdf

[28] Saunders B , Sim J , Kingstone T , et al. Saturation in qualitative research: exploring its conceptualization and operationalization. Qual Quant. 2018;52(4):1893‐1907. 10.1007/s11135-017-0574-8 29937585 PMC5993836

[29] Vasileiou K , Barnett J , Thorpe S , Young T . Characterising and justifying sample size sufficiency in interview‐based studies: systematic analysis of qualitative health research over a 15‐year period. BMC Med Res Methodol. 2018;18(1):148. 10.1186/s12874-018-0594-7 30463515 PMC6249736

[30] Fereday J , Muir‐Cochrane E . Demonstrating rigor using thematic analysis: a hybrid approach of inductive and deductive coding and theme development. Int J Qual Methods. 2006;72:663‐688.

[31] O'Connor DB , Thayer JF , Vedhara K . Stress and health: a review of psychobiological processes. Annu Rev Psychol. 2021;72:663‐688. 10.1146/annurev-psych-062520-122331 32886587

[32] Schneiderman N , Ironson G , Siegel SD . Stress and health: psychological, behavioral, and biological determinants. Annu Rev Clin Psychol. 2005;1:607‐628. 10.1146/annurev.clinpsy.1.102803.144141 17716101 PMC2568977

[33] King L , Rahman M , Wong BC , Mai C , Gould LH . *Health of Asians and Pacifc Islanders in New York City*. NYC Health; 2021, p. 1‐52. https://www.nyc.gov/assets/doh/downloads/pdf/episrv/asian-pacific-islander-health-2021.pdf

[34] Lundy De La Cruz N , Jessup J . *Health of Black New Yorkers by Country of Birth*. New York City Department of Health and Mental Hygiene: Epi Data Brief. 2016. https://www.nyc.gov/assets/doh/downloads/pdf/epi/databrief79.pdf

[35] NCAI Policy Research Center . *Disaggregating American Indian & Alaska native data: a review of the literature*. National Congress of American Indians; 2016.

[36] United States Census Bureau . About the topic of race. 2022. Accessed February 12, 2024. https://www.census.gov/topics/population/race/about.html

[37] Whaley AL , Francis K . Behavioral health in multiracial adolescents: the role of Hispanic/Latino ethnicity. Public Health Rep. 2006;121(2):169‐174. 10.1177/003335490612100211 16528950 PMC1525269

[38] Maghbouleh N , Schachter A , Flores RD . Middle Eastern and North African Americans may not be perceived, nor perceive themselves, to be White. Proc Natl Acad Sci USA. 2022;119(7):e2117940119. 10.1073/pnas.2117940119 35131945 PMC8851556

[39] Awad GH , Kia‐Keating M , Amer MM . A model of cumulative racial–ethnic trauma among Americans of Middle Eastern and North African (MENA) descent. Am Psychol. 2019;74:76‐87. 10.1037/amp0000344 30652901

[40] Rozenkrantz L , D'Mello AM , Gabrieli JDE . Enhanced rationality in autism spectrum disorder. Trends Cogn Sci. 2021;25(8):685‐696. 10.1016/j.tics.2021.05.004 34226128

[41] Ziegler S , Pedersen ML , Mowinckel AM , Biele G . Modelling ADHD: a review of ADHD theories through their predictions for computational models of decision‐making and reinforcement learning. Neurosci Biobehav Rev. 2016;71:633‐656. 10.1016/j.neubiorev.2016.09.002 27608958

[42] Michaels E , Thomas M , Reeves A , et al. Coding the Everyday Discrimination Scale: implications for exposure assessment and associations with hypertension and depression among a cross section of mid‐life African American women. J Epidemiol Community Health. 2019;73(6):577‐584. 10.1136/jech-2018-211230 30894420 PMC7200149

[43] Sue DW , Capodilupo CM , Torino GC , et al. Racial microaggressions in everyday life: implications for clinical practice. Am Psychol. 2007;62(4):271‐286. 10.1037/0003-066X.62.4.271 17516773

[44] Williams DR , Lawrence JA , Davis BA . Racism and health: evidence and needed research. Annu Rev Public Health. 2019;40:105‐125. 10.1146/annurev-publhealth-040218-043750 30601726 PMC6532402

[45] Atkin A . Multiracial identities and resilience to racism: the role of families. January 18, 2023. Accessed February 12, 2024. https://www.medicalnewstoday.com/articles/multiracial-identities-and-resilience-to-racism-the-role-of-families

[46] Espinosa A , Tikhonov A , Ellman LM , Kern DM , Lui F , Anglin D . Ethnic identity and perceived stress among ethnically diverse immigrants. J Immigr Minor Health. 2018;20(1):155‐163. 10.1007/s10903-016-0494-z 27680747 PMC5955603

[47] Forrester S , Jacobs D , Zmora R , Schreiner P , Roger V , Kiefe CI . Racial differences in weathering and its associations with psychosocial stress: the CARDIA study. SSM Popul Health. 2019;7:100319. 10.1016/j.ssmph.2018.11.003 PMC6595283 31294072

[48] Parra LA , Hastings PD . Challenges to identity integration indirectly link experiences of heterosexist and racist discrimination to lower waking salivary cortisol in sexually diverse Latinx emerging adults. Front Psychol. 2020;11:228. 10.3389/fpsyg.2020.00228 32161561 PMC7053485

[49] EHRIntelligence . How health data standards support healthcare interoperability. March 8, 2022. Accessed February 12, 2024. https://ehrintelligence.com/features/how-health-data-standards-support-healthcare-interoperability

[50] Cobb RJ , Thomas CS , Laster Pirtle WN , Darity WA . Self‐identified race, socially assigned skin tone, and adult physiological dysregulation: assessing multiple dimensions of “race” in health disparities research. SSM Popul Health. 2016;2:595‐602. 10.1016/j.ssmph.2016.06.007 29349174 PMC5757885

